# [Retracted] Hydroxytyrosol inhibits cholangiocarcinoma tumor growth: An *in vivo* and *in vitro* study

**DOI:** 10.3892/or.2025.8918

**Published:** 2025-05-26

**Authors:** Shuai Li, Zhiyang Han, Yong Ma, Ruipeng Song, Tiemin Pei, Tongsen Zheng, Jiabei Wang, Dongsheng Xu, Xiang Fang, Hongchi Jiang, Lianxin Liu

Oncol Rep 31: 145–152, 2014; DOI: 10.3892/or.2013.2853

Following the publication of the above paper, a concerned reader drew to the Editor's attention that unexpected similarities existed among the β-actin control western blots in Figs. 1B, 3E and 3G, albeit with the protein bands featured in a different orientation, and with different dimensions, in Fig. 3E compared with Figs. 1B and 3E. Furthermore, the two leftmost β-actin blots in Fig. 3G appeared to show unexpected similarities to the β-actin blots in Fig. 4F, even though different experimental conditions were reported in these figures.

Following an independent assessment of the apparent errors that had been made in assembling the data in the three affected figures, the Editor of *Oncology Reports* has decided that this article should be retracted from the publication on account of a lack of overall confidence in the presented data. The authors were asked for an explanation to account for these concerns, but the Editorial Office did not receive a reply. The Editor apologizes for any inconvenience that might result from this retraction.

